# Regulation of Small Mitochondrial DNA Replicative Advantage by Ribonucleotide Reductase in *Saccharomyces cerevisiae*

**DOI:** 10.1534/g3.117.043851

**Published:** 2017-07-17

**Authors:** Elliot Bradshaw, Minoru Yoshida, Feng Ling

**Affiliations:** * Chemical Genetics Laboratory, RIKEN, Wako, Saitama 351-0198, Japan; † Core Research for Evolutional Science and Technology, Japan Agency for Medical Research and Development, Tokyo 100-0004, Japan; ‡ Graduate School of Science and Engineering, Saitama University, 338-8570, Japan

**Keywords:** DNA replication, heteroplasmy, mitochondrial mutations, ribonucleotide reductase, suppressive mtDNA

## Abstract

Small mitochondrial genomes can behave as selfish elements by displacing wild-type genomes regardless of their detriment to the host organism. In the budding yeast *Saccharomyces cerevisiae*, small hypersuppressive mtDNA transiently coexist with wild-type in a state of heteroplasmy, wherein the replicative advantage of the small mtDNA outcompetes wild-type and produces offspring without respiratory capacity in >95% of colonies. The cytosolic enzyme ribonucleotide reductase (RNR) catalyzes the rate-limiting step in dNTP synthesis and its inhibition has been correlated with increased petite colony formation, reflecting loss of respiratory function. Here, we used heteroplasmic diploids containing wild-type (rho^+^) and suppressive (rho^−^) or hypersuppressive (HS rho^−^) mitochondrial genomes to explore the effects of RNR activity on mtDNA heteroplasmy in offspring. We found that the proportion of rho^+^ offspring was significantly increased by RNR overexpression or deletion of its inhibitor, SML1, while reducing RNR activity via SML1 overexpression produced the opposite effects. In addition, using Ex Taq and KOD Dash polymerases, we observed a replicative advantage for small over large template DNA *in vitro*, but only at low dNTP concentrations. These results suggest that dNTP insufficiency contributes to the replicative advantage of small mtDNA over wild-type and cytosolic dNTP synthesis by RNR is an important regulator of heteroplasmy involving small mtDNA molecules in yeast.

Eukaryotic cells generally contain multiple copies of mitochondrial DNA (mtDNA), which encodes tRNAs, ribosomal RNAs, and electron transport chain subunits essential for mitochondrial respiratory function. These mitochondrial genomes may exist as a mixture of wild-type and mutant copies within a cell, known as heteroplasmy. Within a heteroplasmic cell, unbalanced replication of wild-type and mutant mtDNA alleles may lead to changes in heteroplasmy level, causing deleterious effects on the health of the host organism (Stewart and Chinnery 2015). Deletion mutations, which can shorten the unit length of an mtDNA molecule by several kilobases, can allow a small genome to be copied more quickly and frequently relative to a full-length molecule and thus may possess a replicative advantage. The accumulation of deleted mtDNAs has been observed in tissue-derived and cultured human cells, mice, nematodes, and yeast (Blanc and Dujon 1980; Cortopassi *et al.* 1992; Melov *et al.* 1995; Diaz *et al.* 2002; Fukui and Moraes 2009). *Saccharomyces cerevisiae* has served as a potent example for the rapid expansion of small mtDNA. Fragments of mtDNA containing an active origin of replication (*ori*) sequence were discovered to be hypersuppressive (HS), as they rapidly replicate during heteroplasmy with wild-type mtDNA and become the major mtDNA allele within a few generations, which causes loss of respiratory function in >95% of colonies (Blanc and Dujon 1980).

The complex nature of mtDNA metabolism in yeast is highlighted by the fact that proteins indispensable for rho^+^ mtDNA replication are not always required for the propagation of rho^−^ mtDNA molecules. The mitochondrial RNA polymerase Rpo41 catalyzes mtDNA transcription, initiates RNA-primed mtDNA replication at *ori* sequences, and is required for the stable maintenance of rho^+^ mtDNA (Greenleaf *et al.* 1986). On the other hand, replication of rho^−^ mtDNA molecules can occur independently of RNA priming regardless of *ori* content (Fangman *et al.* 1990). Since one defining characteristic of HS rho^−^ mtDNA is the possession of an active *ori*, a requirement for Rpo41 for the hypersuppressive phenotype would seem obvious, yet crosses between a *∆rpo41* rho^−^ strain lacking an *ori* sequence with a *∆rpo41* HS rho^−^ strain were discovered to result in strongly biased inheritance in favor of the HS rho^−^ allele (Lorimer *et al.* 1995). The question of how the biased inheritance of HS rho^−^ mtDNA is maintained in the absence of RNA priming from *ori* sequences remained. Similarly, the ssDNA binding protein and yeast mitochondrial nucleoid component, Mgm101 (Meeusen *et al.* 1999), is essential for maintenance of rho^+^ genomes containing *ori* and rho^−^ genomes lacking *ori*. Interestingly, maintenance of HS rho^−^ mtDNA was found to occur even in *∆mgm101 ∆rpo41* double-mutant cells (Zuo *et al.* 2002), strongly suggesting the existence of an alternative mtDNA replication pathway.

The mtDNA recombinase Mhr1 catalyzes homologous pairing of nascent ssDNA ends with circular DNA to form a recombination intermediate in which the 3′-ssDNA tail initiates a rolling-circle mode of replication that produces concatemers, linear mtDNA molecules of multiple-unit length (Ling *et al.* 1995; Ling and Shibata 2002, 2004). Ntg1 is a mitochondrial endonuclease that induces DNA double-stranded breaks (DSBs) at *ori5* in response to oxidative stress and, together with Mhr1, contributes to HS rho^−^ mtDNA replication through the initiation of rolling-circle mtDNA replication (Ling *et al.* 2007; Hori *et al.* 2009). Additionally, Din7 is a mitochondrial 5′ to 3′ exodeoxyribonuclease (Fikus *et al.* 2000) that generates 3′ single-stranded DNA tails and was also shown to promote recombination and replication at *ori5* (Ling *et al.* 2013). The Mhr1-catalyzed recombination-dependent rolling-circle replication (RDR) pathway utilizes DSBs, instead of RNA priming at *ori* sequences. Evidence now suggests that the DSB-mediated form of mtDNA replication may be the predominant form of rho^+^ mtDNA maintenance in budding yeast cells. Blocking mtDNA DSBs by binding of mitochondrial-targeted MmKu, which prevents access by eukaryotic repair factors, triggered rho^0^ formation (Prasai *et al.* 2017). Because DSBs frequently occur at *ori5*, the RDR pathway could have a role in the replicative advantage of HS rho^−^ mtDNA.

The conserved Mec1/Rad53 nuclear checkpoint pathway was the first signaling pathway identified to control mtDNA copy number (Taylor *et al.* 2005). Checkpoint activation slows the cell cycle during S phase (Paulovich and Hartwell 1995) and increases cytosolic dNTP synthesis by ribonucleotide reductase (RNR) complex. RNR catalyzes the rate-limiting step of cellular dNTP synthesis through the conversion of ribonucleoside 5′-diphosphates to deoxyribonucleoside 5′-diphosphates and in yeast mainly consists of a large Rnr1-Rnr1 homodimer containing the allosteric feedback and catalytic sites, and a small Rnr2-Rnr4 heterodimer housing the diferric-tyrosyl radical cofactor required for the reduction reaction (Zhang *et al.* 2006). Control of RNR activity in *S. cerevisiae* occurs at four levels: regulation by the transcriptional repressor Crt1 (Huang *et al.* 1998), prevention of Rnr1p homodimerization by binding of the inhibitor Sml1 (Chabes *et al.* 1999), sequestration of the Rnr2-Rnr4 heterodimer in the nucleus (Zhang *et al.* 2006), and allosteric inhibition on the Rnr1 subunit (Chabes *et al.* 2003).

How the replicative advantage of short mtDNA over wild-type is affected by alterations in the RNR pathway remains unexplored. In this study, we have collected evidence to demonstrate a negative correlation between dNTP synthesis by RNR and the replicative advantage for small moderately suppressive or hypersuppressive mtDNA molecules during heteroplasmy with wild-type mtDNA.

## Materials and Methods

### Yeast transformation

Yeast transformation was performed using the lithium-cesium acetate method (Ito *et al.* 1983) using a High Efficiency Yeast Transformation Kit (MoBiTec GmbH). Cloning and overexpression of RNR1 and SML1 was carried out with the plasmid pVT100U (Westermann and Neupert 2000) containing the 397-bp constitutive ADH promoter. Selection for cells harboring the desired plasmids was carried out on synthetic dropout minus uracil (SD-U) plates.

### Yeast crossing experiments

Parental haploid strains were cultivated separately in rich media at 30° overnight to mid log-phase, using YPGlycerol (yeast extract, peptone, 50mM KH_2_PO_4_, 3% glycerol v/v, pH 6.4) for rho^+^ or YPD (yeast extract, peptone, dextrose) medium for rho^−^ cells. Cell concentrations were counted by hemocytometer and 10^7^ cells from each parental strain were added to 1 ml of YPD medium and crossed for 6 hr at 30°. Mated cells were diluted and spread on synthetic defined minimal medium plus leucine (SD+L) or leucine and uracil (SD+LU) agar plates to select for diploid cells (Supplemental Material, Figure S1). Diploid selection plates were incubated at 30° for 2 d and photographed with a LAS-4000 imaging system (GE Healthcare). The diploid selection plates were then replica-plated onto YPGlycerol plates, which were incubated for another 2 d at 30° and then photographed. Images of the SD master plate and its respective YPGlycerol plate were overlaid in Adobe Photoshop Elements and colonies were counted to determine the percentage of rho^+^ colony-forming units (CFUs) formed.

### Quantification of mtDNA levels in heteroplasmic cells

Diploid colonies obtained during crossing experiments were pooled by elution from diploid selection plates with 1× PBS. Samples were pelleted and stored at −80°. Whole yeast DNA including mtDNA was prepared and DNA concentration was measured with a NanoVue spectrophotometer (GE Healthcare). Ninety-five nanograms of whole yeast DNA was used as the standard template concentration for PCR. Primers used for specific detection of nuclear, rho^+^, or HS rho^−^ mtDNA, respectively, were: NUC1-Fwd, 5′-GATACTCTTGTCCGGTTTAGTCG-3′; NUC1-Rev, 5′-ATCTTTCGACTGTTTGATCGCC-3′; COX3-Fwd, 5′-ATGCCTTCACCATGACCTATTG-3′; COX3-Rev, 5′-CCAACATGATGTCCAGCTGTTA-3′; HSC1-Fwd, 5′-GAAGATATCCGGGTCCCAATAATAA-3′; HSC1-Rev, 5′-AATATAATAGTCCCCACTCCGCG-3′. Gels were photographed with a FAS-IV gel imaging system (Nippon Genetics) and band intensities were measured with ImageJ software. MtDNA level was calculated relative to the nuclear DNA signal by the 2^−∆CT^ method (Livak and Schmittgen 2001).

### Western blotting

Parental rho^+^ and HS rho^−^ haploid yeast strains expressing the plasmids pVT100U-Empty, pVT100U-RNR1-FLAG, or pVT100U-SML1-FLAG were grown on SD-U selection plates. Diploid cells were obtained under conditions identical to the crossing experiments and selectively grown to mid log-phase by transferring 20 µl of mated cells to SD+L liquid medium and cultivating overnight at 30°. Protein extraction was performed by the LiAc/NaOH method on ice (Zhang *et al.* 2011). Ten microliters of protein extracts were run on 10 or 15% PAGE gels and semidry transferred to Immobilon-P transfer membranes (Millipore). Primary antibodies used for protein detection were anti-FLAG M2 (Sigma-Aldrich) and yeast anti-Phosphoglycerate Kinase (Invitrogen).

### PCR assay for template amplification rates under increasing dNTP concentrations

Templates used in the PCR assay were generated by inserting tdTomato (Clontech) between the *Kpn*I and *Xba*I cutting sites, or RNR1 between the *Kpn*I and *Xho*I cutting sites of the plasmid pUC119. Template DNA concentrations were optimized to give signals of nearly equivalent apparent strength under ethidium bromide (EtBr) staining. All PCR reactions were performed with *Sca*I-linearized pUC119 templates for 12 cycles. The set of primers flanking the multi-cloning site of plasmid pUC119 used for all PCR reactions was: pUC119-MCS-Fwd, 5′-TTGTGTGGAATTGTGAGCGG-3′; pUC119-MCS-Rev, 5′-TGCAAGGCGATTAAGTTGGG-3′. KOD Dash (TOYOBO) or Ex Taq (Takara Bio.) polymerases were used for PCR. Gels were photographed with a FAS-IV gel imaging system (Nippon Genetics) and band intensities were measured with ImageJ software. All band intensities were normalized against the control band with the weakest EtBr signal (left side of gel; amplified with 200 µM dNTPs). Relative DNA levels (EtBr signal %) at each dNTP concentration were calculated as A_norm_/A_norm_ + B_norm_.

### Microscopy

Parental haploid cells were cultivated in SD-U liquid medium overnight then transferred to rich media and grown to mid log-phase at 30° on a rotary shaker at 120 rpm for 2–6 hr, using YPGlycerol (pH 6.9) for rho^+^ or YPD medium for rho^−^ cells. Cells were treated with 1 µg/ml DAPI and incubated at 30° for 15 min. DAPI-stained cells were then mixed at a 1:1 ratio with 1% low-melting agarose (Lonza), mounted on glass slides and observed with a Deltavision microscope system (Applied Precision) equipped with an IX71 microscope (Olympus). Mitochondria were tagged with GFP for parental rho^−^ cells using the plasmid pVT100U-mtGFP (Westermann and Neupert 2000), or pVT100U-mtTomato, which was constructed by cloning tdTomato (Clontech) into pVT100U, for parental rho^+^ cells.

### Data availability

Yeast strains used in this study are listed in Table S1 and are available upon request.

## Results

### Sml1 is required for the hypersuppressive phenotype

**Figure 1:**
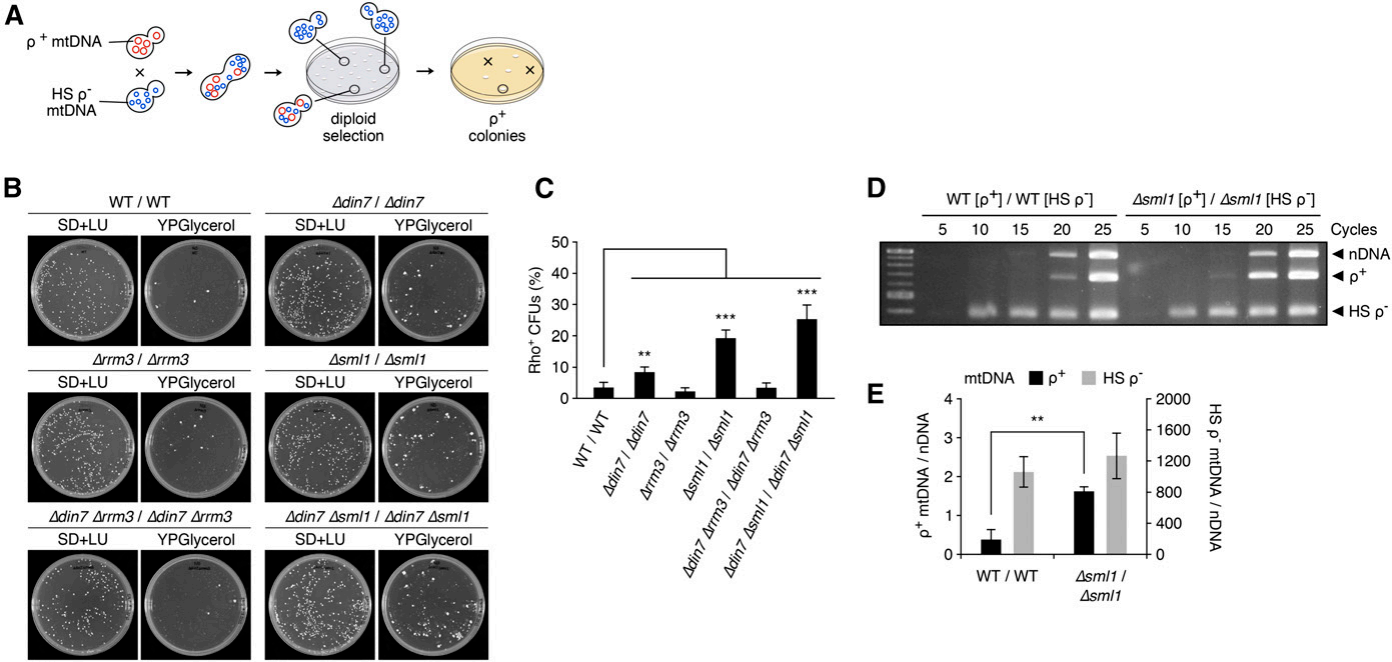
SML1 deletion increases the proportion of rho^+^ colonies and the relative amount of rho^+^ mtDNA during heteroplasmy with hyper-suppressive mtDNA. (A) Scheme of hypersuppressive crossing experiments. Diploid colonies selectively grown on synthetic media contain different amounts of parental rho^+^ and HS rho^−^ mtDNA. Following replica plating, diploid colonies with sufficient rho^+^ mtDNA content are able to grow on YPGlycerol media. (B) Representative images of master and replica plates from genetic crossing experiments. (C) Quantified results of crossing experiments for the genotypes: WT/WT (*n* = 7); *∆din7*/*∆din7* (*n* = 3); *∆rrm3*/*∆rrm3* (*n* = 3); *∆sml1*/*∆sml1* (*n* = 6); *∆din7∆rrm3*/*∆din7∆rrm3* (*n* = 3); *∆din7∆sml1*/*∆din7∆sml1* (*n* = 3). (D) PCR amplified nuclear and mitochondrial DNA from WT/WT and *∆sml1*/*∆sml1* heteroplasmic cells collected from master plates 2 d after crossing. (E) Rho^+^ and HS rho^−^ mtDNA levels were calculated relative to nDNA signals. Quantified mtDNA levels are from three independent PCR experiments. Error bars indicate SD. ** *P* <0.005, *** *P* <0.0005.

To investigate the possible effects of checkpoint signaling on heteroplasmy with HS rho^−^ mtDNA and the involvement of the Mhr1 pathway, we crossed parental strains with the mutations: *∆din7*, *∆rrm3*, *∆sml1*, *∆din7 ∆rrm3*, and *∆din7 ∆sml1*. Heteroplasmic diploids containing the 85.7-kbp wild-type (rho*^+^*) and 1.1-kbp hypersuppressive (HS rho^−^) mitochondrial genomes were produced after mating (Figure 1A) as previously described (Ling *et al.* 2007). The *∆sml1* diploids displayed a marked increase in the proportion of heteroplasmic rho^+^ CFUs, from 3.5 ± 1.7% in wild-type cells to 19.2 ± 2.6% and 25.3 ± 4.5% in *∆sml1* and *∆din7 ∆sml1* mutants, respectively (Figure 1, B and C). *∆din7* cells showed a small increase to 8.5 ± 1.6%, while *∆rrm3* cells gave a slight decrease in rho^+^ CFU formation to 2.3 ± 1.1%, suggesting that neither lack of mitochondrial 5′ to 3′ exonuclease activity nor checkpoint activation induced by nuclear replication-fork stalling, respectively, strongly affected the replicative advantage of HS rho^−^ mtDNA.

To rule out effects from altered mitochondrial morphology or mtDNA nucleoid size in *∆sml1* cells, which could potentially affect mtDNA transmission (Lockshon *et al.* 1995; Westermann 2010), we tagged mitochondria in rho^+^ parental cells with tdTomato and in HS rho^−^ cells with GFP, and stained mtDNA nucleoids with DAPI. We did not observe any apparent differences in mitochondrial morphology or nucleoid size among wild-type and *∆sml1* cells of the same parental background (Figure S2), indicating that the phenotype of *∆sml1* cells is not likely due to irregular transmission of mitochondria or nucleoids.

We detected the proportional amounts of rho^+^ and HS rho^−^ mtDNA in heteroplasmic cells using PCR primers specific for a nuclear gene, rho^+^ mtDNA and HS rho^−^ mtDNA (Figure S3). Analysis of total genomic DNA including mtDNA, obtained by washing all colonies from diploid selection plates, revealed that relative to nuclear DNA (nDNA), levels of rho^+^ mtDNA were 0.38 ± 0.26-fold in wild-type cells compared with 1.64 ± 0.12-fold in *∆sml1* cells (Figure 1, D and E), indicating that the *∆sml1* mutation significantly increased the proportional level of rho^+^ mtDNA in heteroplasmic cells containing HS rho^−^ mtDNA. On the other hand, we observed no significant difference in HS rho^−^ mtDNA levels among the wild-type and *∆sml1* backgrounds, indicating that replication of wild-type mtDNA is increased to a greater extent than HS rho^−^ mtDNA in *∆sml1* cells.

To address the possibility that the increased rho^+^ phenotype was unique to crosses involving our HS rho^−^ strain (YKN1423-C1), we conducted additional crossing experiments with two additional rho^−^ strains: another HS rho^−^ strain (YKN1423-A1) and a moderately suppressive rho^−^ strain (YKN1423-A2). In A1 wild-type crosses, 4.4 ± 0.9% of CFUs were rho^+^ while A1 *∆sml1* crosses gave rise to 75.8 ± 7.8% rho^+^ CFUs. A2 wild-type crosses yielded 66.4 ± 4.0% rho^+^ CFUs while A2 *∆sml1* crosses yielded 79.6 ± 8.1% rho^+^ CFUs (Figure S4, A and B). Attempts to find unique restriction sites in these rho^−^ genomes as a necessary step for sequencing and designing specific PCR primers were unsuccessful. However, we did observe significant increases in rho^+^ mtDNA content in diploids from both crosses (Figure S4, C and D). Taken together, the *∆sml1* mutation increased wild-type mtDNA levels in heteroplasmic cells containing either moderately suppressive or hypersuppressive mtDNA.

### RNR1 overexpression enhances rho^+^ mtDNA replication in hypersuppressive crosses

RNR1 overexpression is sufficient to rescue the temperature-sensitive mtDNA loss phenotype of mitochondrial DNA polymerase *mip1-1* mutants, demonstrating a close relationship between RNR activity and mtDNA maintenance (Lecrenier and Foury 1995). Both RNR1 overexpression and the *∆sml1* mutation increase cellular dNTP concentration (Zhao *et al.* 1998; Chabes and Stillman 2007) and mtDNA copy number (Taylor *et al.* 2005; Lebedeva and Shadel 2007). Furthermore, the *∆sml1* mutation was shown to reduce rates of spontaneous petite formation (Zhao *et al.* 1998). Sml1 inhibits RNR by binding and preventing Rnr1 homodimerization (Chabes *et al.* 1999; Chabes and Stillman 2007), therefore we hypothesized that increased dNTP synthesis by RNR was responsible for the observed increases in rho^+^ CFU formation.

**Figure 2:**
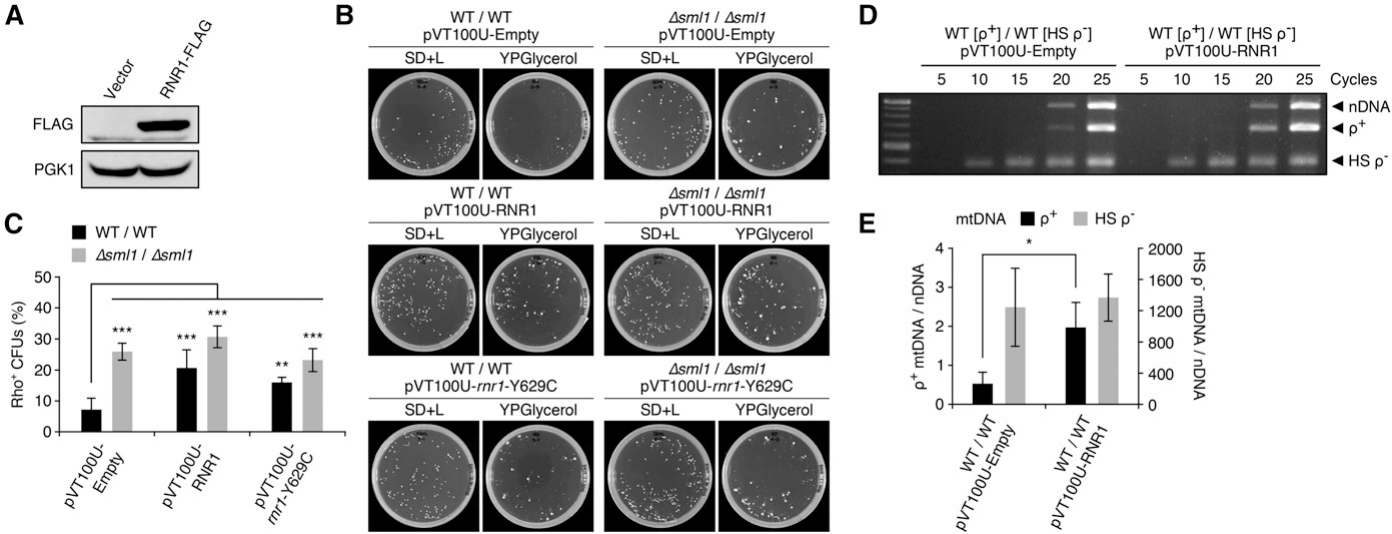
RNR1 overexpression increases rho^+^ mtDNA levels and respiratory function in heteroplasmic cells. (A) Immunoblot of RNR1-FLAG overexpression in heteroplasmic diploid cells following crossing. Anti-PGK is shown as a loading control. (B) Representative images of master and replica plates from genetic crossing experiments of strains expressing an empty vector, overexpressing RNR1, or the mutant *rnr1-Y629C* isoform. (C) Quantified crossing results were obtained from multiple crossing experiments yielding the genotypes: WT/WT/pVT100U-Empty (*n* = 14); *∆sml1*/*∆sml1*/pVT100U-Empty (*n* = 3); WT/WT/pVT100U-RNR1 (*n* = 12); *∆sml1*/*∆sml1*/pVT100U-RNR1 (*n* = 3); WT/WT/pVT100U-*rnr1-Y629C* (*n* = 3); *∆sml1*/*∆sml1*/pVT100U-*rnr1-Y629C* (*n* = 3). (D) PCR amplified nuclear and mitochondrial DNA from WT/WT heteroplasmic cells expressing pVT100U-Empty or pVT100U-RNR1, collected 2 d after crossing. (E) Rho*^+^* and HS rho^−^ mtDNA levels were calculated relative to nDNA signals. Quantified mtDNA levels are from four independent PCR experiments. Error bars indicate SD. * *P* <0.05, ** *P* <0.005, *** *P* <0.0005.

To confirm the role of elevated dNTP synthesis, we overexpressed RNR1 via plasmid and confirmed by immunoblot analysis (Figure 2A). In agreement with the behavior of *∆sml1* crosses, we observed that 20.6 ± 5.9% of heteroplasmic diploid CFUs overexpressing RNR1 were rho^+^, compared with 7.2 ± 3.7% of diploid CFUs expressing the empty vector (Figure 2, B and C). In the strain containing the empty vector, levels of rho^+^ mtDNA were 0.53 ± 0.29-fold relative to NUC1, compared with 1.97 ± 0.64-fold in the RNR1-overexpressing strain (Figure 2, D and E). There was no significant change in levels of HS rho^−^ mtDNA among cells expressing the empty or RNR plasmids. In addition, we expressed a mutant isoform, *rnr1*-Y629C, and observed that 15.9 ± 1.7% and 23.2 ± 3.7% of wild-type and *∆sml1* diploid CFUs, respectively, were rho^+^ in *rnr1*-Y629C expressing cells. The lower rho^+^ CFU formation rate suggests a lower catalytic activity of the *rnr1*-Y629C mutant gene product and supports the notion that elevated RNR activity contributes to the replication of rho^+^ genomes in the presence of HS rho^−^ mtDNA, consistent with the observation of cells lacking Sml1.

### Overproducing Sml1 in ∆sml1 cells restores the hypersuppressive phenotype

**Figure 3:**
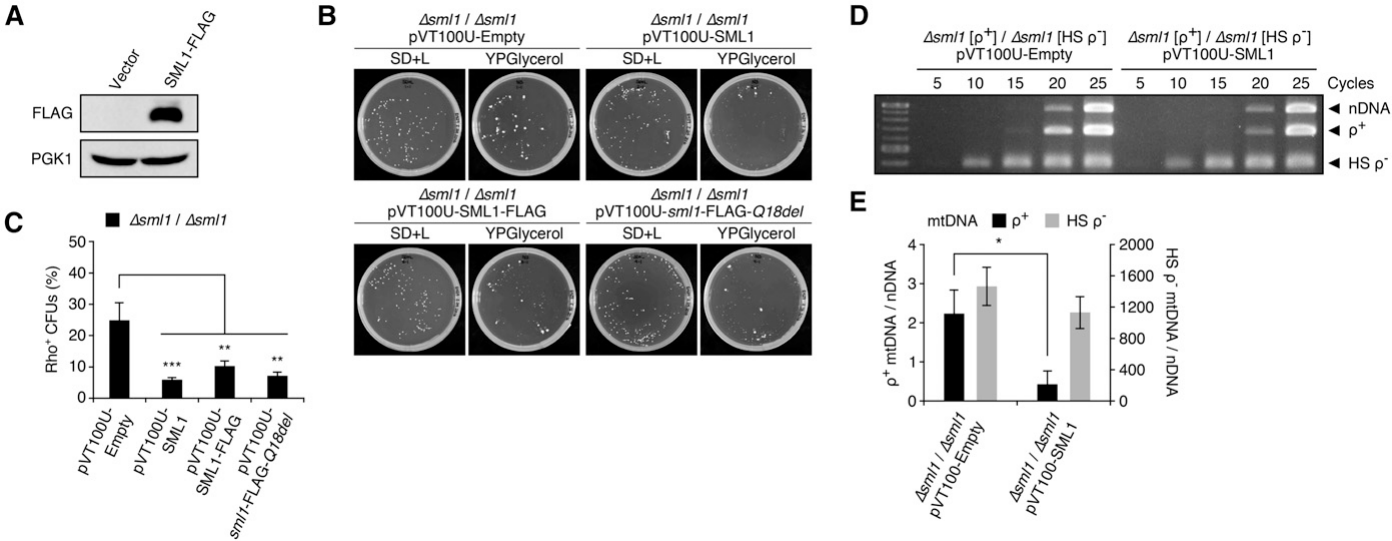
SML1 overexpression suppresses rho^+^ mtDNA levels and respiratory function in heteroplasmic cells. (A) Immunoblot of SML1-FLAG overexpression in heteroplasmic diploid cells following crossing. Anti-PGK is shown as a loading control. (B) Representative images of master and replica plates from genetic crossing experiments of strains expressing an empty vector, or overexpressing SML1, SML1-FLAG, or the mutant *sml1*-FLAG*-Q18del* isoform. (C) Quantified crossing results were obtained from multiple crossing experiments yielding the genotypes: *∆sml1*/*∆sml1*/pVT100U-Empty (*n* = 8); *∆sml1*/*∆sml1*/pVT100U-SML1 (*n* = 8); *∆sml1*/*∆sml1*/pVT100U-SML1-FLAG (*n* = 3); *∆sml1*/*∆sml1*/pVT100U-*sml1*-FLAG*-Q18del* (*n* = 3). (D) PCR amplified nuclear and mitochondrial DNA from *∆sml1*/*∆sml1* heteroplasmic cells expressing pVT100U-Empty or pVT100U-SML1, collected 2 d after crossing. (E) Rho^+^ and HS rho^−^ mtDNA levels were calculated relative to nDNA signals. Quantified mtDNA levels are from three independent PCR experiments. Error bars indicate SD. * *P* <0.05, ** *P* <0.005, *** *P* <0.0005.

Sml1 inhibits RNR outside of S phase when demand for dNTP synthesis is low and is removed during S phase or in response to DNA damage (Zhao *et al.* 2001; Chabes *et al.* 2003). SML1 overexpression increases the frequency of spontaneous petite colony formation compared to wild-type cells, indicating that mitochondrial genome maintenance is impaired by the inhibition of cytosolic dNTP synthesis by RNR (Zhao *et al.* 1998). To further demonstrate the relationship between the RNR pathway and selfish mtDNA dynamics, we examined whether artificially lowering RNR activity by increasing its inhibition can restore the replicative advantage of small hypersuppressive mtDNA. We confirmed SML1 overexpression by immunoblot (Figure 3A) and found a significant decrease in the proportion of rho^+^ CFUs from 24.9 ± 5.6% in *∆sml1* cells containing the empty vector to 5.9 ± 0.7%, 10.3 ± 1.6%, and 7.2 ± 1.1% in *∆sml1* cells overexpressing SML1, SML1-FLAG, and *sml1-FLAG-Q18del*, respectively (Figure 3, B and C). Since the Sml1 protein consists of only 111 amino acids, the relative size of the FLAG tag may have lowered its binding and inhibitory effect on Rnr1p, while the Q18 deletion appears to have slightly improved inhibitory function. Consistent with the drop in rho^+^ CFU formation rate, rho^+^ mtDNA level declined approximately fivefold, from 2.23 ± 0.61-fold relative to NUC1 in *∆sml1* cells expressing the empty plasmid to 0.43 ± 0.34-fold upon SML1 expression (Figure 3, D and E). On the other hand, we observed a small but not statistically significant decrease in the level of HS rho^−^ mtDNA level upon SML1 expression. Together, these results indicate that artificially lowering RNR activity enhances the replicative advantage of hypersuppressive over wild-type mtDNA.

### Low dNTP concentration enhances the replicative advantage of small template DNA over large in vitro

**Figure 4:**
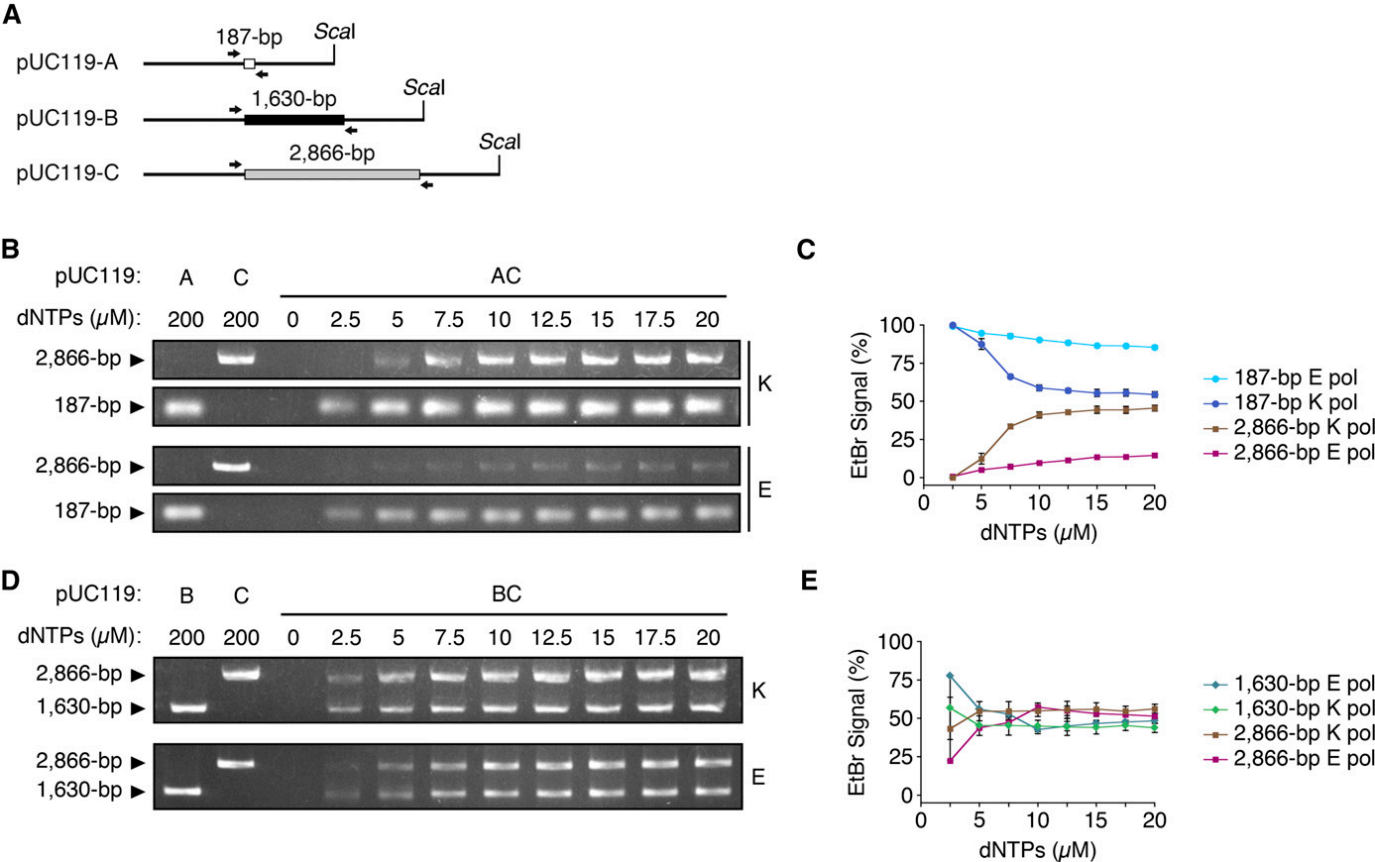
Competitive amplification of DNA templates of disparate lengths under increasing dNTP concentrations *in vitro*. (A) Schematic of *Sca*I-linearized pUC119 DNA templates. (B) DNA amplification following 12 PCR cycles with templates A and C in isolation at a dNTP concentration of 200 µM (left), or a mixture of pUC119-A and pUC119-C at dNTP concentrations from 0 to 20 µM. K, KOD Dash polymerase; E, Ex Taq polymerase. (C) Percentage of the total signal representing the relative amount of the 187- or 2866-bp template amplified at the indicated dNTP concentrations. (D) DNA amplification following 12 PCR cycles with templates B and C in isolation at a dNTP concentration of 200 µM (left), or a mixture of pUC119-B and pUC119-C at dNTP concentrations from 0 to 20 µM. K, KOD Dash polymerase; E, Ex Taq polymerase. (E) Percentage of the total signal representing the relative amount of the 2866- or 1630-bp template amplified at the indicated dNTP concentrations. Results in C and E represent three independent experiments using KOD Dash polymerase and two independent experiments using Ex Taq polymerase. Error bars indicate SD.

Overexpression of RNR1 or the *∆sml1* mutation are known to positively regulate dNTP concentration and mtDNA copy number in yeast, and our experimental results suggest that relatively low dNTP concentration may contribute to the replicative advantage of small mtDNA. To further illustrate the effect of low dNTP concentration, we tested competitive amplification by PCR using Ex Taq or KOD Dash polymerases and templates of different size (Figure 4A). We examined a dNTP concentration range of 0 to 20 µM, as these levels reflect the physiological dNTP concentrations within mammalian mitochondria (Song *et al.* 2005).

Consistent with our observations of suppressive mtDNA in yeast crossing experiments, the small template was amplified much more readily compared to the large at dNTP concentrations of <10 µM (Figure 4, B and C and Figure S5, A and B). The strength of this effect varied between the two polymerases tested; however, the replicative advantage of the smallest template decreased with increasing dNTP concentration and signals from either large PCR product (2866 or 1630-bp) did not exceed those of the small (187-bp) PCR product at any dNTP concentration. On the other hand, during PCR amplification of two templates of closer size, the small template only displayed a replicative advantage at 2.5 µM with one of the two polymerases under our experimental conditions (Figure 4, D and E). Additionally, PCR reactions using a mixture of all three templates showed that the smallest template was amplified almost exclusively at dNTP concentrations of <7.5 µM (Figure S5, C and D). These data show that dNTP concentration and the relative sizes of templates are important factors in replicative advantage during PCR.

## Discussion

**Figure 5:**
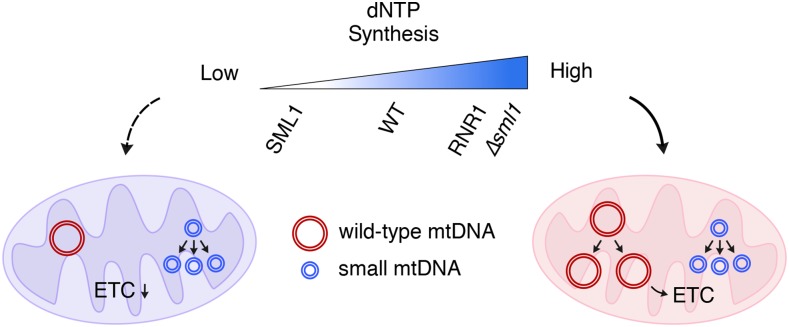
Model for the role of cytosolic dNTP synthesis in regulating rho^+^ mtDNA replication during heteroplasmy with small mtDNA in yeast. In heteroplasmic cells containing a mixture of small and full-length mtDNA, the wild-type or SML1-overexpressing backgrounds have dNTP synthesis levels only sufficient for small mtDNA replication, causing most cells to lose respiratory function. Increased dNTP synthesis by RNR1 overexpression or SML1 deletion allows more full-length mtDNA replication, resulting in improved respiratory growth via electron transport chain function.

Disruption of dNTP balance or availability within mitochondria has been linked to mtDNA depletion and disease (Gonzalez-Vioque *et al.* 2011; Dalla Rosa *et al.* 2016) and promotes mtDNA deletion mutagenesis in cultured cells (Song *et al.* 2005). In this study, we demonstrated that the replicative advantage of moderately suppressive or hypersuppressive mtDNA molecules is partially due to insufficient dNTP synthesis by RNR. Competition between small and full-length mtDNA in heteroplasmic cells is naturally weighted against a larger allele; however, reducing RNR activity appears to enhance the replicative advantage of small mtDNA. Indeed, competitive amplification of a mixture of small and large templates via PCR showed that the replicative advantage of small DNA is affected by relative template size and dNTP concentration *in vitro*. Though replication *in vivo* by mtDNA polymerase γ occurs under physiological conditions and in conjunction with the mitochondrial replisome, both mtDNA polymerase γ and Taq polymerases are derived from the A family of DNA polymerases (Steitz 1999), while KOD enzymes belong to family B (Elshawadfy *et al.* 2014), which possess a catalytic “palm” domain homologous to family A polymerases. Both PCR enzymes showed a general trend of increasing replicative advantage for smaller templates as dNTP concentrations decrease. Taken together, these results support a model wherein dNTP synthesis by RNR influences the extent of the replicative advantage of small mtDNA in yeast, and therefore affects mtDNA heteroplasmy level and respiratory function (Figure 5).

Nuclear DNA damage in yeast activates the Mec1/Rad53 nuclear checkpoint pathway, halts the cell cycle (Paulovich and Hartwell 1995), and increases dNTP production through the removal of Sml1 (Zhao *et al.* 2001; Chabes *et al.* 2003) and increased transcription of the RNR genes (Huang *et al.* 1998). Importantly, checkpoint activation was also shown to increase mtDNA copy number as much as twofold in *∆rrm3* and *∆sml1* deletion mutants (Taylor *et al.* 2005). However, as shown in Figure 1, the *∆rrm3* mutation did not increase the proportion of rho^+^ CFUs formed during heteroplasmy with HS rho^−^ mtDNA, suggesting that increased mtDNA point mutagenesis in the *∆rrm3* background (O’Rourke *et al.* 2005) may strongly affect heteroplasmic cells.

We previously showed that the Mhr1 pathway regulates DSB-induced RDR in response to oxidative stress. The *ori5* region is particularly sensitive to oxidative modification, and following exposure to hydrogen peroxide, Ntg1 was shown to increase DSB formation in this locus (Ling *et al.* 2007; Hori *et al.* 2009). Supporting this notion, the DSB-binding protein MmKu binds preferentially to the *ori5* region, though *∆ntg1* cells showed only a slight decrease in MmKu binding, indicating that additional factors likely contribute to DSB formation at *ori5* (Prasai *et al.* 2017). DSBs are substrates of Din7, which catalyzes 5′ end resection to yield 3′-ssDNA tails, which can then be used for homologous pairing by Mhr1 to initiate RDR (Ling *et al.* 2013). Compared to 85.7-kbp wild-type mtDNA, the 1.1-kbp HS [*ori5*] rho^−^ mtDNA molecule has a much higher density of *ori5* sequences. Therefore, reducing Mhr1 pathway activity through the *∆din7* mutation could be expected to disproportionately inhibit replication of HS [*ori5*] rho^−^ mtDNA compared to wild-type. In our experiments, crosses of *din7* null mutants did show a small but significant (*P* = 0.002) increase in the rho^+^ CFU formation rate compared to wild-type crosses. However, due to the presence of a functional SML1 gene, any benefit for wild-type mtDNA synthesis in the *∆din7* background was likely reduced due to the suppression of dNTP synthesis. Indeed, the *∆din7 ∆sml1* background showed an additive effect compared with *∆din7* (*P* = 0.003) or *∆sml1* (*P* = 0.032) single-mutants, indicating that dNTP availability plays an important regulatory role in the replicative advantage of HS rho^−^ mtDNA.

Ribonucleotide reductase is considered an attractive target for inhibiting cell proliferation in cancer therapy and other disease. Here, our study in yeast suggests that inhibiting dNTP synthesis may produce the undesirable effect of increasing the replicative advantage of small mtDNAs, which have been associated with aging and several aging-related diseases in humans (see review by Kauppila *et al.* 2017). Precisely how RNR may contribute to human aging and disease in this context remains for future study.

## Supplementary Material

Supplemental material is available online at www.g3journal.org/lookup/suppl/doi:10.1534/g3.117.043851/-/DC1.
