# Supplementary material for: Regulation of Small Mitochondrial DNA Replicative Advantage by Ribonucleotide Reductase in Saccharomyces cerevisiae
Source: G3 (Bethesda). 2017 Jul 17;7(9):3083–90. doi: 10.1534/g3.117.043851 (PMC5592933; doi:10.1534/g3.117.043851)
Supplement: Supplementary file 1 [file 3083FigureS1.pdf]

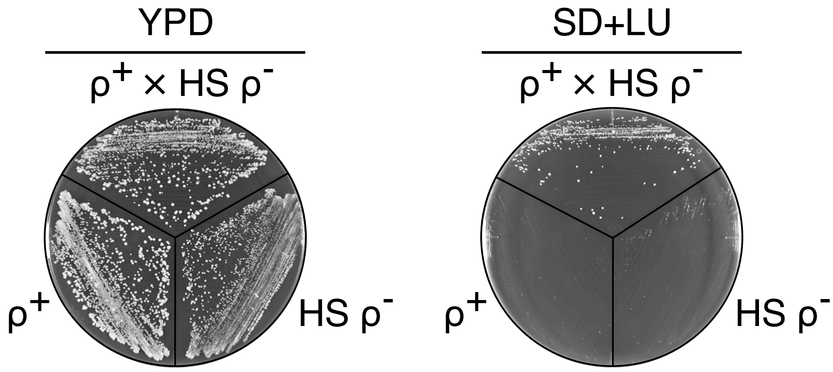

Figure S1 Validation of diploid selection. Rho^+ and HS rho^- parental haploid cells and diploid cells mated for six hours were spread across YPD (left) and SD+LU (right) agar plates.
