# Supplementary material for: Regulation of Small Mitochondrial DNA Replicative Advantage by Ribonucleotide Reductase in Saccharomyces cerevisiae
Source: G3 (Bethesda). 2017 Jul 17;7(9):3083–90. doi: 10.1534/g3.117.043851 (PMC5592933; doi:10.1534/g3.117.043851)
Supplement: Supplementary file 2 [file 3083FigureS2.pdf]

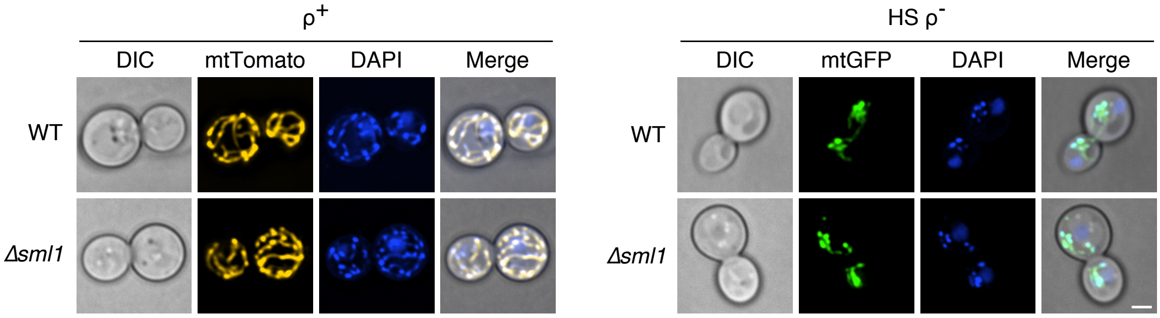

Figure S2 Microscopic examination of mitochondrial morphology and nucleoids in parental haploid cells. Mitochondria in WT and Δ*sml1* parental haploid cells were visualized by expression of mtTomato (rho^{+} cells) or mtGFP (HS rho^{-} cells) from the plasmid pVT100U. Mitochondrial nucleoids were visualized by DAPI staining. Scale bar = 2 µm.
