# Supplementary material for: Regulation of Small Mitochondrial DNA Replicative Advantage by Ribonucleotide Reductase in Saccharomyces cerevisiae
Source: G3 (Bethesda). 2017 Jul 17;7(9):3083–90. doi: 10.1534/g3.117.043851 (PMC5592933; doi:10.1534/g3.117.043851)
Supplement: Supplementary file 3 [file 3083FigureS3.pdf]

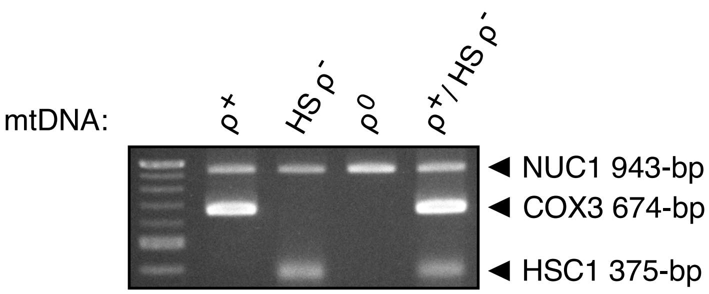

Figure S3 Primer specificity for examination of relative rho$^+$ and HS rho$^-$ mtDNA levels in heteroplasmic cells. Total genomic DNA including mtDNA was prepared from W303a-187 (rho$^+$), YKN1423C-1 (HS rho$^-$), IL166-5b (rho^0) and OP11c-55R5 (rho$^+$) / YKN1423C-1 (HS rho$^-$) cells. Each genomic DNA sample was amplified for 20 PCR cycles in three separate reactions with the primer sets: NUC1 (nuclear DNA); COX3 (rho$^+$ mtDNA); HSC1 (HS rho$^-$ mtDNA). For the respective cell samples, 3 μl of each PCR reaction product was mixed and run in a 1.8% agarose gel (pictured).
