# Supplementary material for: Regulation of Small Mitochondrial DNA Replicative Advantage by Ribonucleotide Reductase in Saccharomyces cerevisiae
Source: G3 (Bethesda). 2017 Jul 17;7(9):3083–90. doi: 10.1534/g3.117.043851 (PMC5592933; doi:10.1534/g3.117.043851)
Supplement: Supplementary file 4 [file 3083FigureS4.pdf]

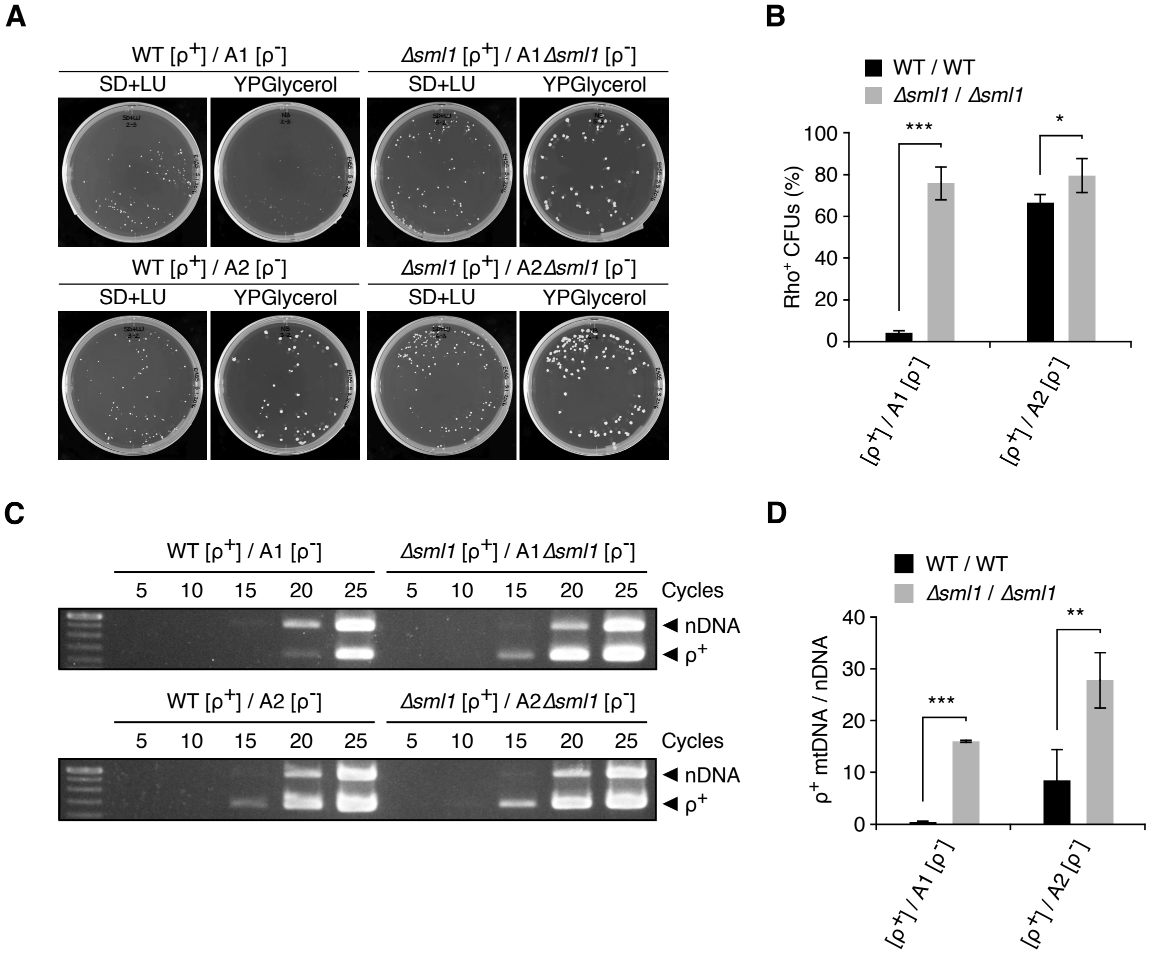

Figure S4 Effect of SML1 deletion on heteroplasmic cells containing different hypersuppressive rho$^-$ and normal suppressive rho$^-$ alleles. (A) Representative images of master and replica plates from genetic crossing experiments. (B) Quantified results of crossing experiments for the genotypes: WT/A1 WT (*n* = 3); Δ*sml1*/A1 Δ*sml1* (*n* = 3); WT/A2 WT (*n* = 4); Δ*sml1*/A2 Δ*sml1* (*n* = 4). (C) PCR amplified nuclear and mitochondrial DNA from WT/A1 WT and Δ*sml1*/A1 Δ*sml1* (top) or WT/A2 WT and Δ*sml1*/A2 Δ*sml1* heteroplasmic cells (bottom). (D) Rho$^+$ (COX3) mtDNA levels were calculated relative to nuclear DNA (NUC1) signals. Quantified results were obtained from independent crossing experiments for WT/A1 WT (*n* = 3); Δ*sml1*/A1 Δ*sml1* (*n* = 3); WT/A2 WT (*n* = 4); and Δ*sml1*/A2 Δ*sml1* (*n* = 4) cells. Error bars indicate SD. *, $P < 0.05$; **, $P < 0.005$; ***, $P < 0.0005$.
