# Supplementary material for: Regulation of Small Mitochondrial DNA Replicative Advantage by Ribonucleotide Reductase in Saccharomyces cerevisiae
Source: G3 (Bethesda). 2017 Jul 17;7(9):3083–90. doi: 10.1534/g3.117.043851 (PMC5592933; doi:10.1534/g3.117.043851)
Supplement: Supplementary file 5 [file 3083FigureS5.pdf]

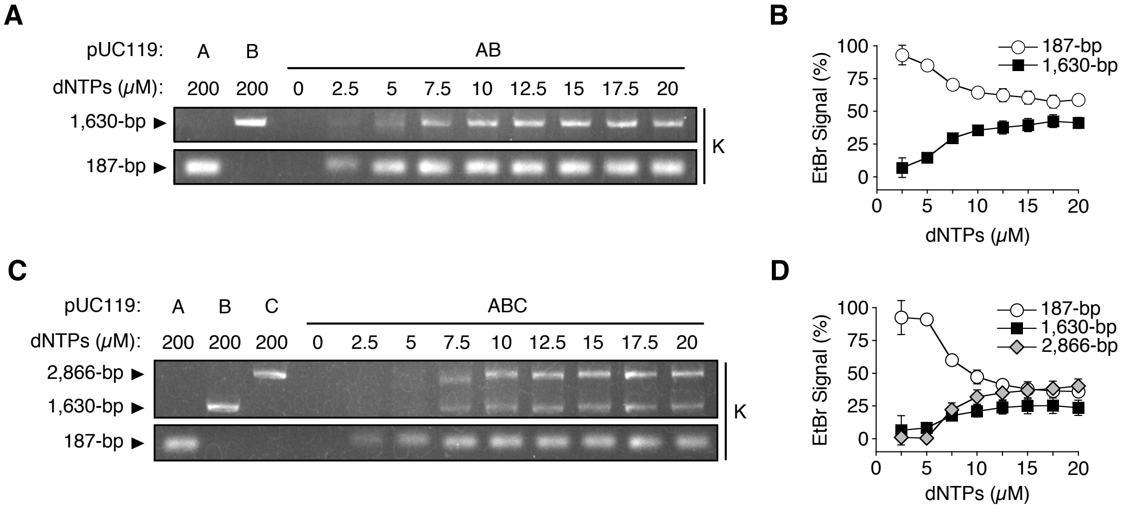

Figure S5 Extended data for competitive amplification of DNA templates of disparate lengths under increasing dNTP concentrations *in vitro.* (A) DNA amplification following 12 PCR cycles with templates A and B in isolation at a dNTP concentration of 200 µM (left), or a mixture of pUC119-A and –B at dNTP concentrations from 0 to 20 µM. K: KOD Dash polymerase. (B) Percentage of the total signal representing the relative amount of the 187-bp or 1,630-bp template amplified at the indicated dNTP concentrations. (C) DNA amplification following 12 PCR cycles with templates A, B and C in isolation at a dNTP concentration of 200 µM (left), or a mixture of pUC119-A, -B and –C at dNTP concentrations from 0 to 20 µM. K: KOD Dash polymerase. (D) Percentage of the total signal representing the relative amount of the 187-bp, 1,630-bp or 2,866-bp template amplified at the indicated dNTP concentrations.
