# Supplementary material for: Regulation of Small Mitochondrial DNA Replicative Advantage by Ribonucleotide Reductase in Saccharomyces cerevisiae
Source: G3 (Bethesda). 2017 Jul 17;7(9):3083–90. doi: 10.1534/g3.117.043851 (PMC5592933; doi:10.1534/g3.117.043851)
Supplement: Supplementary file 6 [file 3083TableS1.pdf]

Supplemental Table 1. Yeast strains used in this study

**Strain**	**Nuclear genotype**	**Mitochondrial genotype**	**Source**
OP11c-55R5	*MAT***a** *leu2 ura3 trp1*	[ρ^{+} $\boldsymbol{\omega}^{-}$ *ens*2 Oli_{1}^{R}]	Ling *et al.* (38)
OP11c-55R5/pVT100U	*MAT***a** *leu2 ura3 trp1* pVT100U (URA3)	[ρ^{+} $\boldsymbol{\omega}^{-}$ *ens*2 Oli_{1}^{R}]	This study
OP11c-55R5/pVT100U-mtTomato	*MAT***a** *leu2 ura3 trp1* pVT100U (mtTomato, URA3)	[ρ^{+} $\boldsymbol{\omega}^{-}$ *ens*2 Oli_{1}^{R}]	This study
OP11c-55R5/pVT100U-RNR1	*MAT***a** *leu2 ura3 trp1* pVT100U (RNR1, URA3)	[ρ^{+} $\boldsymbol{\omega}^{-}$ *ens*2 Oli_{1}^{R}]	This study
OP11c-55R5/pVT100U-*rnr1*-Y629C	*MAT***a** *leu2 ura3 trp1* pVT100U (*rnr1*, URA3)	[ρ^{+} $\boldsymbol{\omega}^{-}$ *ens*2 Oli_{1}^{R}]	This study
OP11c-55R5/pVT100U-SML1	*MAT***a** *leu2 ura3 trp1* pVT100U (SML1, URA3)	[ρ^{+} $\boldsymbol{\omega}^{-}$ *ens*2 Oli_{1}^{R}]	This study
OP11c-55R5 Δ*sml1*/pVT100U	*MAT***a** *leu2 ura3 trp1 sml1::KAN* pVT100U (URA3)	[ρ^{+} $\boldsymbol{\omega}^{-}$ *ens*2 Oli_{1}^{R}]	This study
OP11c-55R5 Δ*sml1*/pVT100U-mtTomato	*MAT***a** *leu2 ura3 trp1 sml1::KAN* pVT100U (mtTomato, URA3)	[ρ^{+} $\boldsymbol{\omega}^{-}$ *ens*2 Oli_{1}^{R}]	This study
OP11c-55R5 Δ*sml1*/pVT100U-RNR1	*MAT***a** *leu2 ura3 trp1 sml1::KAN* pVT100U (RNR1, URA3)	[ρ^{+} $\boldsymbol{\omega}^{-}$ *ens*2 Oli_{1}^{R}]	This study
OP11c-55R5 Δ*sml1*/pVT100U-*rnr1*-Y629C	*MAT***a** *leu2 ura3 trp1 sml1::KAN* pVT100U (*rnr1*, URA3)	[ρ^{+} $\boldsymbol{\omega}^{-}$ *ens*2 Oli_{1}^{R}]	This study
OP11c-55R5 Δ*din7*	*MAT***a** *leu2 ura3 trp1 din7::URA3*	[ρ^{+} $\boldsymbol{\omega}^{-}$ *ens*2 Oli_{1}^{R}]	This study
OP11c-55R5 Δ*din7* Δ*rrm3*	*MAT***a** *leu2 ura3 trp1 din7::URA3 rrm3::KAN*	[ρ^{+} $\boldsymbol{\omega}^{-}$ *ens*2 Oli_{1}^{R}]	This study
OP11c-55R5 Δ*din7* Δ*sml1*	*MAT***a** *leu2 ura3 trp1 din7::URA3 sml1::KAN*	[ρ^{+} $\boldsymbol{\omega}^{-}$ *ens*2 Oli_{1}^{R}]	This study
OP11c-55R5 Δ*rrm3*	*MAT***a** *leu2 ura3 trp1 rrm3::KAN*	[ρ^{+} $\boldsymbol{\omega}^{-}$ *ens*2 Oli_{1}^{R}]	This study
OP11c-55R5 Δ*sml1*	*MAT***a** *leu2 ura3 trp1 sml1::KAN*	[ρ^{+} $\boldsymbol{\omega}^{-}$ *ens*2 Oli_{1}^{R}]	This study
YKN1423A-1	*MATα leu2 ura3 met3*	[HS ρ^{-}]	Ling *et al.* (13)
YKN1423A-1 Δ*sml1*	*MATα leu2 ura3 met3 sml1::KAN*	[HS ρ^{-}]	This study
YKN1423A-2	*MATα leu2 ura3 met3*	Normal suppressive [ρ^{-}]	Ling *et al.* (13)
YKN1423A-2 Δ*sml1*	*MATα leu2 ura3 met3 sml1::KAN*	Normal suppressive [ρ^{-}]	This study
YKN1423C-1	*MATα leu2 ura3 met3*	[HS ρ^{-}]	Ling *et al.* (13)
YKN1423C-1/pVT100U	*MATα leu2 ura3 met3* pVT100U (URA3)	[HS ρ^{-}]	This study
YKN1423C-1/pVT100U-mtGFP	*MATα leu2 ura3 met3* pVT100U (mtGFP, URA3)	[HS ρ^{-}]	This study
YKN1423C-1/pVT100U-RNR1	*MATα leu2 ura3 met3* pVT100U (RNR1, URA3)	[HS ρ^{-}]	This study
YKN1423C-1/pVT100U-*rnr1*-Y629C	*MATα leu2 ura3 met3* pVT100U (*rnr1*, URA3)	[HS ρ^{-}]	This study
YKN1423C-1 Δ*sml1*/pVT100U	*MATα leu2 ura3 met3 sml1::KAN* pVT100U (URA3)	[HS ρ^{-}]	This study
YKN1423C-1 Δ*sml1*/pVT100U-mtGFP	*MATα leu2 ura3 met3 sml1::KAN* pVT100U (mtGFP, URA3)	[HS ρ^{-}]	This study
YKN1423C-1 Δ*sml1*/pVT100U-RNR1	*MATα leu2 ura3 met3 sml1::KAN* pVT100U (RNR1, URA3)	[HS ρ^{-}]	This study
YKN1423C-1 Δ*sml1*/pVT100U-*rnr1*-Y629C	*MATα leu2 ura3 met3 sml1::KAN* pVT100U (*rnr1*, URA3)	[HS ρ^{-}]	This study
YKN1423C-1 Δ*din7*	*MATα leu2 ura3 met3 din7::URA3*	[HS ρ^{-}]	This study
YKN1423C-1 Δ*din7* Δ*rrm3*	*MATα leu2 ura3 met3 din7::URA3 rrm3::KAN*	[HS ρ^{-}]	This study
YKN1423C-1 Δ*din7* Δ*sml1*	*MATα leu2 ura3 met3 din7::URA3 sml1::KAN*	[HS ρ^{-}]	This study
YKN1423C-1 Δ*rrm3*	*MATα leu2 ura3 met3 rrm3::KAN*	[HS ρ^{-}]	This study
YKN1423C-1 Δ*sml1*	*MATα leu2 ura3 met3 sml1::KAN*	[HS ρ^{-}]	This study
W303a-187	*MAT***a** *ade2 leu2 his3 ura3 trp1 can1*	[ρ^{+} $\boldsymbol{\omega}^{-}$ *ens*2 Chl_{321}^{R}]	Ling *et al.* (12)
IL166-5bρ^{0}	*MATα his1 trp1 can1*	[ρ^{0}]	Derived from IL166-187 by EtBr treatment
